# The Development of Teacher Burnout and the Effects of Resource Factors: A Latent Transition Perspective

**DOI:** 10.3390/ijerph19052725

**Published:** 2022-02-26

**Authors:** Min Xie, Shunsen Huang, Li Ke, Xia Wang, Yun Wang

**Affiliations:** 1 State Key Laboratory of Cognitive Neuroscience and Learning, Beijing Normal University, Beijing 100875, China; 201731430010@mail.bnu.edu.cn (M.X.); huangss@mail.bnu.edu.cn (S.H.); 2 Collaborative Innovation Center of Assessment for Basic Education Quality, Beijing Normal University, Beijing 100875, China; ke@bnu.edu.cn; 3 Beijing Institute of Education Science, Beijing 100080, China; wangxia620620@163.com

**Keywords:** teacher burnout, latent profiles, latent transition analysis, resource factors

## Abstract

To better understand burnout and its development, researchers have shown an increasing interest in recent years in identifying different profiles of burnout and its development process. However, there have been few longitudinal studies on the profile and development of teacher burnout. This study used a person-centred approach to explore the profiles of teacher burnout, transition probabilities and the associations between these aspects and resource factors. Data were collected from 3743 primary school teachers in a two-wave longitudinal test over three years. The results showed that teacher burnout exhibited six relatively stable profiles across the whole study population and that the transition of individual profiles over time followed a certain probability. Psychological capital and professional identity were important resource factors in reducing the occurrence of teacher burnout and increasing transition probability toward burnout symptom alleviation over time, while positive coping played an important role in reducing the occurrence of teacher ineffectiveness. Therefore, the results indicated that the overall teacher burnout profile was stable, a discovery which has important implications for conducting group interventions to benefit more teachers, while the individual burnout profile exhibited a latent transition probability over time. Interventions employing different resource factors can be adopted to alleviate the symptoms of different burnout profiles.

## 1. Introduction

Teacher burnout is a psychological syndrome that teachers experience in response to chronic job stress, and includes emotional exhaustion (EE), depersonalization (DP), and reduced personal accomplishment (PA) [1]. EE refers to feelings of overextending and draining emotional resources, while DP refers to negative, callous, or unfeeling responses to the job, and PA refers to feelings of incompetency and reduction in productivity. There are many direct, mediating, and moderating factors that contribute to teacher burnout [2,3,4,5,6,7,8,9,10]. However, there have been few longitudinal studies on teacher burnout, and the developmental aspects of teacher burnout remain controversial [11]. Theoretically, there are five prominent models that describe the developmental process of burnout symptoms [11,12,13,14,15], but they remain debated in terms of theory and lack consistent findings. One reason for this lack of findings may be that such studies have ignored the fact that individuals in the same dataset follow different processes of burnout [16,17]. Leiter and Maslach (2016) [16] argued that dividing burnout into different profiles could provide a better understanding of burnout and its development processes, and they proposed evaluating the relative stability of various profiles through longitudinal studies. However, very few studies have investigated the profiles of teacher burnout and their relative stability using longitudinal data [8]. A three-wave longitudinal study divided teacher burnout into seven developmental categories based only on the emotional exhaustion dimension of burnout [18]. These studies focused on the different development of individual burnout profiles but ignored the question of whether the whole sample exhibited the same burnout profiles at different points in time, that is, the relative stability of the burnout profiles themselves. To attain a more profound understanding of the profiles and development of teacher burnout, this study explores the question of whether the whole sample exhibits the same profiles at varying points in time and investigates the transformation of individual profiles across times.

Latent profile analysis (LPA), as a person-centred approach, fits well with these multidimensional profiles [16,17]. LPA grants the capacity to identify the ideal number of latent subgroups (profiles) in a population based on an individual’s response to multiple observed variables [19]. Latent transition analysis (LTA) is an extension of LPA in which the probabilities of transitions from each class at one point in time to all others at the next point in time are estimated using longitudinal data [20]. LPA and LTA are part of a person-centred analytical approach that aims to identify subgroups of individuals with similar scores on various indicators of interest [21].

This study aimed to complement previous person-centred teacher burnout research and contribute to the teacher burnout literature in three important ways. First, longitudinal data was used to capture possible profiles of teacher burnout and to examine the relative stability of these profiles over time. Based on the theoretical model of burnout development at the whole level (e.g., the eight-phase model of [12]), the burnout profiles may be the same from an overall perspective. If the latent profiles of teacher burnout were found to be stable at different points in time, it would be more conducive to understanding and discovering the characteristics of teacher burnout theoretically and it would be of great significance for making and popularizing the targeted interventions in practice, which has not been mentioned by previous studies. Second, via longitudinal data, the understanding of the development of teacher burnout was enhanced by considering the possibility that the burnout profiles would not transfer in the same direction across all individuals. In other words, this study considered both the overall consistency of teacher burnout profiles and the variability of teacher burnout profiles at the individual level. Third, the effect of individual resources (here, psychological capital, professional identity and positive coping) was taken into account on the disposition of teacher burnout profiles and the latent transition of different profiles over time. These resource factors were confirmed to be important for teacher burnout [6,9,22]. Our study has the potential to produce a variety of information concerning the link between individual resources and burnout profiles and their roles in the transition of different burnout profiles, which is valuable for practical work.

## 2. Theoretical Background

### 2.1. Development and Profiles of Burnout

Burnout has been studied in the field of psychology and education for over forty years, and teacher burnout has been an important part of burnout research [1]. It is helpful to review the research regarding the development and profiles of burnout to guide the study of teacher burnout. To date, two lines of research have been conducted to examine the development and profiles of burnout. The first line of research has employed a variable-centred approach (e.g., regression-based analysis) to explore the development of burnout across the whole study population [23]. This line of research has made a valuable contribution to the long-term interplay of three burnout symptoms. At least five prominent models have described the sequential process of the three symptoms of burnout [11,12,13,14,15], although thus far, none of these models has proved to be preferable [24,25,26,27]. However, this line of research has ignored the various types of burnout and the fact that the development of burnout may follow different trajectories. In recent years, a second line of research has been developed. This line of research uses a person-centred approach (e.g., median splits, or latent profile analysis) to capture the profile features of burnout and its development within an individual [8,16,17,28]. These studies have confirmed that the development of burnout does not follow a uniform pattern and that there may be different burnout profiles at varying times. However, these studies have not explored whether the same burnout profiles exist at different points in time throughout the whole study population. For example, Golembiewski et al. (1983) [12] described eight burnout profiles at the group level using a median split based on their developmental model at the individual level. They declared that different individuals exhibited different burnout profiles, and that not every individual had to experience all profiles. In other words, individuals may exhibit different profiles of burnout at varying times, but overall, the same profiles of the three burnout symptoms persist.

For the teacher population, only a few studies have investigated the development and profiles of teacher burnout, especially in the context of longitudinal studies. For example, Taris et al. (2005) [11] compared several developmental models in a longitudinal study referencing a sample of teachers and proposed the Taris (2005) model, which was “EE→DP→EE&PA”, for the whole study population, but the authors did not describe the profiles of teacher burnout. Méndez et al. (2020) [8] divided teacher burnout into three profiles based on a cross-sectional study, including profiles that were low in three symptoms, high in three symptoms, and low in EE and DP but high in reduced PA. However, no study has analysed both teacher burnout profiles and their development in tandem with the three burnout symptoms using longitudinal data. Exploring more detailed information concerning the profiles of teacher burnout and the transitions among them would facilitate interventions for subgroups of teachers with different profiles. This study explored the questions of whether the whole sample of teachers exhibits the same profiles at different points in time and how individual profiles transfer over time using the LPA/LTA method. Theoretically, in the case of three symptoms, a developmental model could empirically construct eight profiles (high or low in each symptom × three symptoms, 8 = 2^3^) using the median split method. As noted by Golembiewski et al. (1983) [12], middling profiles were not easy to distinguish. The LPA method may incorporate some middling profiles to improve the differentiation of latent profiles [16]. The hypotheses are as follows:

**Hypothesis** **1.** *Teacher burnout exhibits the same profiles across time throughout the whole study population. Specifically, based on development models, there are no more than eight profiles, and the three dimensions of burnout are well distinguished.*

**Hypothesis** **2.** *In different burnout profiles, the burnout profiles of individuals transfer to other profiles with a certain transition proportion over time. Given the interactions among different situational factors and different individual cognitions, the transition probabilities from the same burnout profile to other burnout profiles are unequal.*

### 2.2. Individual Resource Factors and Teacher Burnout

Classrooms, schools, and society all demand more from teachers without providing proportional resources [5]. However, this situation does not mean that burnout symptoms will continue to worsen as long as the stress exists. The conservation of resources theory (COR theory) [29,30] and the job demand-resource model (JD-R model) [31,32] indicate that sufficient and effective resources play an important role in alleviating the negative impact of job stress on burnout. Here, psychological capital, professional identity, and positive coping are considered to be important individual resources to combat stress and reduce burnout. Psychological capital is defined as a positive psychological state of development that supplements an individual’s energy to regulate behaviour [33,34]. Professional identity is defined as individuals’ own recognition of their specific occupational interests, abilities, goals, and values [35]. Positive coping refers to the specific effort that an individual makes to master, reduce, tolerate or minimize stressful events [3].

There is substantial empirical evidence that these three resource factors are linked to the reduction of burnout and its symptoms. For example, psychological capital can supplement the energy of individuals to regulate their behaviour and reduce the onset of burnout [34]. Psychological capital has not only significantly negative effects on the three symptoms of teacher burnout [6,9] but is also an important moderator on the effect of risk factors (e.g., occupational stress, emotional labour, and work-family conflict, etc.) on teacher burnout [7,36,37]. According to social identity theory, individuals need to exhibit self-identification to establish a positive self-image [38]. Teachers with low professional identity lack affirmation of their professional value, and therefore they tend to feel irritable and indifferent about their work, while teachers with high professional identity tend to exhibit low levels of burnout [10]. Coping can be understood from a stylistic point of view [39] and involves a set of cognitive and behavioural strategies. Positive coping, which refers to taking a direct and rational approach to dealing with the problem, is an important predominant predictor of low levels of teacher burnout [6,40,41]. Furthermore, some researchers have reported that positive coping is an important mediator in the relationship between psychological capital and teacher burnout [6]. However, no study has examined the effects of the three resource factors on the development and profile of teacher burnout. Therefore, this study examined the influence of psychological capital, professional identity, and positive coping on the disposition of the profiles of teacher burnout and the transitions among them. The hypothesis is as follows:

**Hypothesis** **3.** *Psychological capital, professional identity, and positive coping affect the occurrence probability of the profiles of teacher burnout and the transition probability among different profiles.*

## 3. Materials and Methods

### 3.1. Participants

Two-waves of data concerning primary school teachers in Beijing were collected. Excluding teachers who retired within three years, 3743 teachers participated in the first wave (T1) in November 2014, while 3247 (86.7%) teachers participated in the second wave (T2) in November 2017. Little’s MCAR (missing completely at random) test showed that the missing data were MCAR (χ^2^ = 125.647, *df* = 107, *p* = 0.105), and missing values were estimated using the full-information maximum likelihood (FIML) procedure [42]. At T1, 18.9% of teachers were male, and the percentages of teachers with less than 10 years, 10–20 years and more than 20 years of teaching experience were 22.3%, 35.7%, and 42%, respectively; the percentage of teachers in charge of classes was approximately 53.3%; the percentages of teachers who were married, unmarried, and divorced/widowed were approximately 87.4%, 9.7%, and 2.9%, respectively; and the percentages of teachers who did not teach or taught grade 1, grade 2, grade 3, grade 4, grade 5 and grade 6 were 3.8%, 14.2%,15.2%, 16.2%, 17.6%, 15.7%, and 17.2%, respectively.

### 3.2. Instruments

#### 3.2.1. Teacher Burnout

Based on the Chinese Teacher Job Burnout Inventory developed by Wang and Xu (2004) [43], members of our group interviewed in-service teachers from primary and secondary schools and then made certain modifications to the items. The revised burnout scale included three dimensions, namely emotional exhaustion (EE, nine items), depersonalization (DP, six items) and reduced personal accomplishment (PA, four items), which were rated by teachers on a 5-point Likert scale ranging from 1 (never) to 5 (always). At T1, the internal consistency coefficient was α = 0.91, and the construct validity indices were CFI = 0.93, TLI = 0.92, RMSEA = 0.07, SRMR = 0.05; at T2, these measures were α = 0.93, CFI = 0.94, TLI = 0.93, RMSEA = 0.07, SRMR = 0.05, all indicating good and robust reliability and validity.

#### 3.2.2. Psychological Capital

The Psychological Capital Questionnaire (PCQ) [44] was used to assess the psychological capital of teachers. Some expressions were adapted to suit the teaching profession (e.g., by modifying “company” to “school”). The PCQ included four subscales: hope (six items), resilience (six items), optimism (six items), and efficacy (six items). The test items were rated on a 4-point Likert scale ranging from 1 (strongly disagree) to 4 (strongly agree). In this study, the Cronbach’s α was 0.94, CFI = 0.90, and TLI = 0.88, indicating good internal consistency reliability and construct validity.

#### 3.2.3. Professional Identity

Professional identity was assessed using the Teachers’ Professional Identity Scale [35]. This scale included four dimensions, occupational values (4 items), role values (6 items), sense of occupational belonging (3 items), and professional behaviour inclination (5 items). Each item was rated on a 5-point Likert scale ranging from 1 (completely disagree) to 5 (completely agree). In this study, Cronbach’s α was 0.96, CFI = 0.92, TLI = 0.90, indicating good internal consistency reliability and construct validity.

#### 3.2.4. Positive Coping Style Questionnaire

Primary and secondary teachers with different teaching experiences were interviewed based on the Chinese version of the Coping Style Questionnaire developed by Xiao & Xu (1996) [45], and then certain modifications were made. The revised questionnaire consisted of four dimensions: positive problem solving, seeking help from others, positive view of the problem, and reasonable catharsis, with 15 items rated on a 4-point Likert scale ranging from 1 (never use) to 4 (always use). The reliability and validity of the revised questionnaire were α = 0.89, CFI = 0.88, TLI = 0.86 among primary school teachers, which demonstrated good reliability.

All instruments were measured in accordance with teachers’ self-evaluation, and a test of common method bias was implemented using the Harman single-factor method. The results showed that a total of 14 factors with characteristic roots greater than 1 were extracted. The interpretation rate of the first factor was 32.26%, lower than the critical standard of 40%, indicating the absence of serious common method bias [46].

### 3.3. Procedure

The survey of teachers’ working status was commissioned to be conducted by the education administration of a district in Beijing. Prior to the survey, all schools and teachers in the district were briefed on the purpose of the survey and the benefits they would receive at the end of the survey (e.g., report of results, etc.), and were invited to participate voluntarily. The online assessment system remained open for one week, and the process was anonymous, voluntary, and confidential. If teachers wanted to participate in this survey, they could complete the test at their convenience by logging into the system from their computer or cell phone and could stop or opt out at any time. The ethical requirements of the subjects were fully considered during the data collection process.

### 3.4. Data Analysis

SPSS 26.0 statistical software (IBM, New York, NY, USA) was used for data management, descriptive statistical analysis and multivariate multinomial logistic regression analysis. Mplus 8 [47] was used for LPA and LTA.

In LPA, data fitting indices included AIC, aBIC, entropy, p(LMR), and p(BLRT). AIC referred to the Akaike information criterion, aBIC indicated the Bayesian information criterion calibrated by sample size, and entropy denoted the average information, which was an indicator of classification accuracy. Entropy ranged from 0 to 1, the closer to 1 the better, while values greater than 0.8 indicated good model fit [48]. p(LMR) referred to the significance level of Lo-Mondell-Ruben’s calibrated likelihood ratio test and p(BLRT) indicated the significance level based on the bootstrap likelihood ratio test.

In descriptive statistical analysis, the mean process was used to compute the mean value and standard deviation of every factor in each latent profile, and the ANOVA was used to analyse the mean difference of all latent profiles. Due to the large sample size of this study, the effect size η^2^ was reported and η^2^ > 0.01 indicated that the difference was significant [49].

In multivariate multinomial logistic regression analysis, compared with the reference profile, the odds ratio (OR) for other profiles under the effect of each factor was reported. When analysing the occurrence probability of each latent profile under the influence of resource factors, the reference group was “Low/no burnout” profile and an OR value greater than 1 meant that, under the influence of this factor, the probability of individuals falling into this profile is greater than that falling into the “Low/no burnout” profile. When analysing the influence of resource factors on the latent transition of each latent profile, the latent profiles at T1 were separated, and there were six logistic models. For each latent profile at T1, the teachers who stayed in the same profile at T2 were the reference group. The OR here referred to the ratio of the probability of transferring to another profile compared with that of staying in the original profile and an OR value greater than 1 indicated that the transition probability increased under the effect of this influencing factor. For each OR value, *p* < 0.05 (expressed in an asterisk) indicated that the OR value was significant. Furthermore, for each factor, there was a *p*-value (the last column) of the likelihood ratio test as a whole and *p* < 0.05 indicated that the factor had a significant influence on the outcome variables.

## 4. Results

### 4.1. Latent Profiles of Burnout

Eight latent profile models were constructed with a number of categories ranging from 1 to 8. Table 1 presents the results of profile enumeration at T1 and T2. As shown in Table 1, the LMR test suggested that the number of profiles did not exceed 7. The three-profile, six-profile and seven-profile solutions were supported by the entropy values at T1, while T2 featured solutions with two to six profiles. The AIC and aBIC values indicated that adding new categories improved model fit. Therefore, the 6- profile solution was best, and the results showed that the six profiles were consistent at T1 and T2. However, the 6-profile model featured a small profile that contained <5% of the sample. Thus, to avoid possible spurious profiles, two five-profile models were analysed and the results showed that the five profiles at T2 were consistent with the five profiles of Leiter & Maslach (2016) [16], but at T1 the five profiles hid the profile “EE&DP dominated burnout” (named “burnout” in Leiter & Maslach, 2016) which exhibited the smallest proportion, and highlighted the profile “highly ineffective” (never mentioned in Leiter & Maslach, 2016) which exhibited a relatively high proportion. That is, the proportion of these two profiles was small, but they both existed. These two profiles stood in opposition to one another and were reasonable in a theoretical sense [12], so the 6-profile solution was retained.

**Figure 1:**
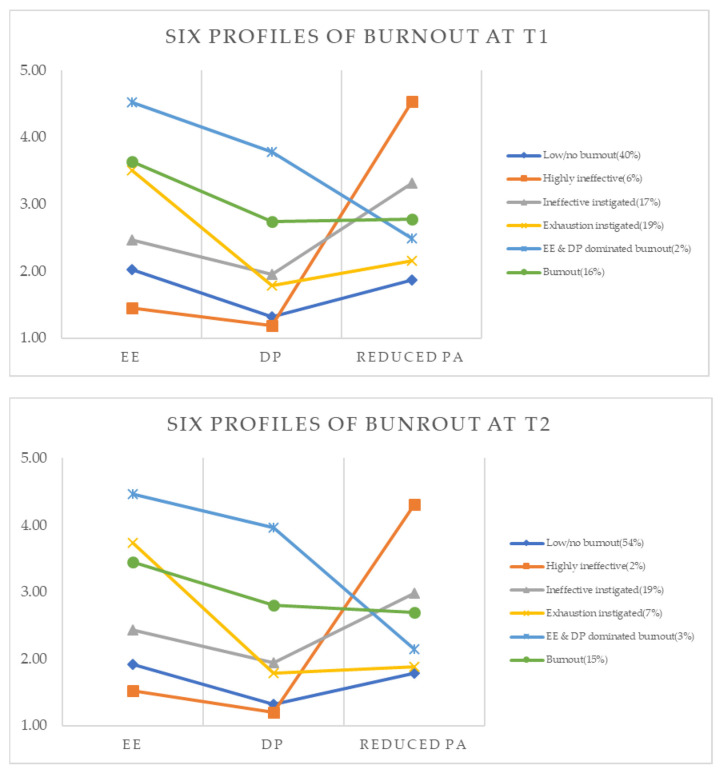
Six latent profiles of burnout at T1 and T2.

Table 2 presents the mean values of each dimension for every profile, and a graphical display of the means can be found in Figure 1. Based on the patterns observed in these figures, we labelled the six profiles as follows: (1) Low/no burnout, (2) Highly ineffective (very high in reduced PA, very low in the other two dimensions), (3) Ineffective instigated (high in reduced PA, moderate in the other two dimensions), (4) Exhaustion instigated (high exhaustion, moderate in the other two dimensions), (5) EE & DP dominated burnout (very high EE and DP, moderate in reduced PA), and (6) Burnout (high in all three dimensions). The variance analysis results (*F* test and effect size η^2^) showed that the differences in the mean of the six profiles at T1 and T2 were all very significant in all three dimensions. In the post test, the differences between any two profiles were significant, indicating that each profile was well distinguished, except for the means of reduced PA between the profile 1 and profile 4 at T2.

### 4.2. Latent Transition among Profiles over Time

Burnout is a dynamic process [12,50], so the burnout profile of the same individual may differ over time. Table 3 presents the latent probabilities of individuals in certain profiles transferring to another profile from T1 to T2. The results showed that “Low/no burnout” individuals at T1 had a latent probability of 62% of maintaining this profile; “Highly ineffective” individuals at T1 had a latent probability of 60.1% of transferring to “Low/no burnout” at T2; “Ineffective instigated” individuals at T1 had a latent probability of 33% of maintaining this profile at T2 and latent probabilities of 24.6% and 21.3% of transferring to “Burnout” and “Highly ineffective”, respectively; “Exhaustion instigated” individuals at T1 had a latent probability of 43.7% of transferring to “Ineffective instigated” at T2 and 27% of maintaining this profile; “EE&DP dominated burnout” individuals had a latent probability of 29.2% of maintaining this profile and 24.3% of transferring to “Burnout” at T2; “Burnout” individuals had a latent probability of 30.9% of maintaining this profile and 23.9% of transferring to “Ineffective instigated” at T2.

### 4.3. The Effect of Resource Factors on Latent Profiles and the Transitions among Them

Table 4 presents the means and standard deviations of the three resource factors in each profile. The variance analysis results showed that the differences in the means of all six profiles with respect to the three resource factors were all very significant. “Low/no burnout” teachers performed best on these three factors. “EE&DP dominated burnout” teachers had the lowest values of psychological capital and professional identity. “Ineffective instigated”, “Burnout” and “Highly ineffective” teachers had relatively low mean values of positive coping.

Table 5 presents the OR for each profile under the effects of psychological capital, professional identity, and positive coping, while individuals in the “Low/no burnout” profile served as the reference group. As shown in Table 5, both psychological capital and professional identity decreased risks of being in the “Ineffective instigated”, “Exhaustion instigated”, “EE&DP dominated burnout”, and “Burnout” profiles. Under the influence of psychological capital or professional identity, compared with the “Low/no burnout” profile, the probabilities of being in these four latent profiles were significantly lower. Unexpectedly, professional identity increased the risk of being “Highly ineffective”. Under the influence of positive coping, the probability of being in the “High ineffective” profile was lower, while the probabilities in the other latent profiles were not significantly different from those in the “Low/no burnout” profile. That is, positive coping played a limited role in establishing individual burnout profiles.

Table 6 presents the OR of the transition from T1 to T2 with the effects of the three factors. The OR here refers to the ratio of the probability of transferring to another group compared with that of staying in the original group. The results showed that psychological capital, professional identity, and positive coping all played important roles in alleviating burnout symptoms among teachers compared with maintaining their current profile.

Under the influence of psychological capital, “Low/no burnout” teachers had a significantly lower probability of transferring to other profiles (all ORs were significantly less than 1), while teachers in other profiles (e.g., Ineffective instigated, Exhaustion instigated and Burnout) had a significantly higher probability of transferring to “Low/no burnout” (all ORs were significantly greater than 1). In addition, “Highly ineffective”, “Ineffective instigated”, and “Exhaustion instigated” teachers had significantly lower probabilities of transferring to “Burnout” (OR = 0.03, 0.52, 0.05, respectively).

The effects of professional identity were similar to those of psychological capital, except for the effects of professional identity on the transitions from “Low/no burnout” and “Ineffective instigate” to “Highly ineffective”. Under the influence of professional identity, “Low/no burnout” and “Ineffective instigate” teachers had a significantly higher probability of transferring to “Highly ineffective”.

Under the influence of positive coping, “Low/no burnout” teachers had a significantly lower probability of transferring to “Ineffective instigated” and “Burnout” at T2 (OR = 0.52, 0.48, respectively). “Highly ineffective”, “Ineffective instigated” and “Burnout” teachers had a significantly higher probability of transferring to “Low/no burnout” (OR = 3.63, 2.20, 2.37, respectively). In addition, “Exhaustion instigated” teachers had a significantly lower probability of transferring to “Highly ineffective” and “Ineffective instigated” (OR = 0.08, 0.45, in turn), while “EE&DP dominated burnout” and “Burnout” teachers had a significantly lower probability of transferring to “Ineffective instigated” (OR = 0.15, 0.54, respectively). All of these transitions suggested that positive coping played an important role in reducing the occurrence of teacher ineffectiveness.

## 5. Discussion

Our study explored the latent profiles of teacher burnout and the question of whether these profiles could be differentiated based on their relations with individual resource factors, such as psychological capital, professional identity, and positive coping. The results showed that there were six consistent latent profiles of teacher burnout with a meaningful difference in the whole study population at T1 and T2 and that teachers in one profile would transfer to another profile with a latent transition probability over time. It was demonstrated that the burnout dynamics and the temporal sequence of burnout symptoms varied across teachers, but that the same profiles were generally maintained on the whole. In addition, psychological capital and professional identity were important influencing factors in reducing the occurrence of teacher burnout and in increasing transition probability toward burnout symptom alleviation over time, while positive coping played an important role in reducing the occurrence of teacher ineffectiveness.

### 5.1. Profiles of Burnout—The Differential Roles of Individual Resource Factors

The present study using the LPA approach confirmed the first hypothesis that the same profiles existed at varying times throughout the whole study population and that the “Low/no burnout” profile accounted for the largest proportion of the sample (see Table 2). Specifically, two-wave longitudinal tests found that there were six well-distinguished stable latent burnout profiles, namely, “Low/no burnout”, “Highly ineffective”, “Ineffective instigated”, “Exhaustion instigated”, “EE&DP dominated burnout” and “Burnout”. The result of six latent profiles was similar to the five profiles developed by Leiter and Maslach (2016) [16]. The difference was that the present study distinguished the “Highly ineffective” profile, although the proportion of this profile was very small. This profile was similar to but different from the “Ineffective instigated” profile. As seen from the results of this study (Table 3), when EE and DP were low, the symptom of highly reduced personal accomplishment was easily alleviated and there was a high latent transition probability to the “Low/no burnout” profile. However, the symptoms of “Ineffective instigated” were relatively difficult to alleviate and had a higher latent probability of transferring to the “Burnout” or “Highly ineffective” profiles. This result suggested that the median split method may obscure some important subtypes. Although these subtypes appeared to be similar, they may lend themselves to different intervention strategies. The ineffective profile (including both “Highly ineffective” and “Ineffective instigated”) reflects a psychological relationship with work, and it suggests that work-life experience was not at the same level of self-actualization. This profile was more prevalent among teachers in both tests (>20%), and it suggested that ineffectiveness (reduced PA) was actually a far more common experience among teachers; even if not yet well understood, this ineffective profile deserves more research attention [8,16,51].

Although the three symptoms of burnout have been widely accepted, their independence and dependence on each other have been debated [50,52,53]. In the present study, the ineffective profile (including “Highly ineffective” and “Ineffective instigated”) and the exhaustion profile (“Exhaustion instigated”) were identified, with the exception of a profile named “Depersonalization instigated” (high DP, low EE and reduced PA). Higher DP appeared only when EE was high (“EE&DP dominated burnout”) or when all three dimensions were high (“Burnout”), not in isolation. Leiter and Maslach (2016) [16] claimed to have discovered this profile, but it has not been found by other studies [17,54], including the profile study of teacher burnout [8]. In studies using the median split method, this profile existed, but in a very small proportion [12]. In this study, the mean value of this dimension was obviously lower than that of the other two dimensions, meaning that most teachers did not think that they treated students negatively and coldly. Whether this manifests differently in different occupations is unclear. However, depersonalization plays an important role in the development of teacher burnout [11], and the question of how to better identify depersonalization among teachers is an issue of concern. The most direct effect of depersonalization was the disruption of interpersonal relationships [55,56], and poor interpersonal relationships may also increase burnout [57,58]. Thus, an alternative way of identifying early symptoms of depersonalization may be to include interpersonal assessments in teacher evaluations.

This study adds to what is known about the impact of individual resource factors on the development process of teacher burnout. The results of this study concurred with the COR theory [29,30] and the JD-R model [31,32], suggesting that individual resources can reduce burnout symptoms. Previous studies have revealed the direct or moderating effects of psychological capital, professional identity, and positive coping on burnout [6,9,10,41,59]; however, the present study contributed to the burnout literature by showing that psychological capital and professional identity are important influencing factors in reducing the occurrence of teacher burnout and increasing the transition probability of each profile toward burnout symptom alleviation over time, while positive coping plays an important role in reducing the occurrence of teacher ineffectiveness.

Specifically, teachers with high psychological capital or high professional identity were more likely to be in the “Low/no burnout” profile and less likely to be in the “Ineffective instigated”, “Exhaustion instigated”, “EE&DP dominated burnout” and “Burnout” profiles. Additionally, during the development of individual burnout profiles, teachers had a significantly higher probability of transferring to “Low/no burnout” and had a significantly lower probability of transferring to “Burnout”. In other words, psychological capital and professional identity could not only reduce the occurrence of various burnout profiles, but also help teachers reduce burnout symptoms and avoid worse situations when burnout occurs. This effect was not obviously associated with different burnout profiles or burnout symptoms. Compared with psychological capital and professional identity, the role of positive coping in reducing the occurrence of various burnout profiles was limited; however, positive coping played an important role in improving the efficacy of teachers. Previous studies have not emphasized this point [6,41]. Of course, most previous studies have analysed the relationship between coping and teacher burnout in cross-sectional data, while no study has analysed the role of positive coping in the development of teacher burnout by using longitudinal data. As mentioned above, depersonalization is a form of dysfunctional coping that reduces the accomplishment of teachers [11]. Could positive coping improve teachers’ accomplishment? In an intervention study on coping strategies and socioemotional competence, participating teachers demonstrated an increase in positive coping strategies and a significant increase in personal accomplishment [41]. More longitudinal studies are needed to analyse the impact of positive coping on teacher effectiveness.However, there were two unexpected findings: professional identity was associated with the “Highly ineffective” profile compared to the “Low/no burnout” profile and was associated with the transition from the “Low/no burnout” profile to the “Highly ineffective” profile compared with staying in the low/no burnout profile. One possibility was that there was a high proportion of male teachers among the “Highly ineffective” profile (34%, compared with 18.9% in the total sample, 17.4% in “Low/no burnout” profile, and 22.7% in “Ineffective instigated” profile). Male teachers were more likely than female teachers to be in the “Highly ineffective” profile (OR = 2.44, *p* = 0.030) and were more likely to transfer from the “Low/no burnout” profile to the “Highly ineffective” profile (OR = 2.82, *p* = 0.048). When gender effects were present, higher professional identity did not increase the probability of teachers being in the “Highly ineffective” profile compared the “Low/ no burnout” profile (OR = 1.34, *p* = 0.232), but it still increased the probability of transition from the “Low/no burnout” profile to the “Highly ineffective” profile (OR = 5.10, *p* = 0.013). The second possibility was that the professional identity of teachers among the “Highly ineffective” profile was too high, even higher than teachers among the “Low/no burnout” profile (Table 4). According to the environment perspective of job burnout, burnout occurs when individual goals and expectations are not successfully translated into actual value [55,60]. However, whether there are differences in burnout developmental trends between male and female teachers among the “Highly ineffective” profile and whether there is a covariant relationship between such trends and professional identity needs to be explored in future longitudinal studies with more time points and a larger sample.

### 5.2. Implications for Interventions

These profiles and the roles of individual resource factors could also have implications for interventions. As burnout interventions and rehabilitation are not always effective in treating severe chronic burnout [61,62], more emphasis needs to be placed on taking proactive measures to prevent burnout, for example, by distinguishing different burnout profiles [16,17] and improving individual resources [63,64].

For example, “Burnout” teachers exhibited low scores on all three resource factors, and an increase in each factor would help alleviate their burnout symptoms. “Ineffective instigated” teachers had high scores on professional identity but low scores on psychological capital and positive coping, so improving psychological capital and positive coping strategies among this group of teachers would be helpful. For “Exhaustion instigated” teachers, their relatively high positive coping scores prevented them from transferring to “Highly ineffective” and “Ineffective instigated” but had no obvious effect on the alleviation of exhaustion. They may need psychological capital intervention training or an increase in their recognition of the value of their teaching. The proportion of teachers who fell into the profiles of “Highly ineffective” and “EE&DP dominated burnout” was very small, and it was more important to distinguish the two profiles. The former group needs an increase in positive coping strategies, while the latter group needs an increase in psychological capital and professional identity. Besides, burnout is a very common issue among teachers, even in the recent context of COVID-19 [65,66]. We believed that our study may provide some suggestions for intervention for teachers suffering from burnout during the pandemic.

There have already been some intervention programs for teachers or other groups that have focused on these three resource factors [41,67,68,69]. We can learn from these intervention programs to solve the problems of the five profiles of teacher burnout (except the “Low/no burnout” profile). However, we suggest that it is desirable to distinguish different burnout profiles before initiating targeted interventions, as burnout interventions are not always effective for different samples [61,62]. In addition, the reduced PA dimension has been more neglected in previous burnout research [16], while in the profile study of burnout, researchers proposed to pay attention to this dimension [16], which may be a more decisive symptom of burnout [17]. Furthermore, the results of this study indicate that no matter how individual burnout profiles transfer over time, there are six relatively stable profiles of teacher burnout across the population for which corresponding intervention measures can be formulated (except the “Low/no burnout” profile). This approach has at least two advantages: one advantage is that doing so improves the effectiveness of intervention and constantly refines the intervention based on the effect of intervention; the other advantage is that doing so facilitates group intervention in one or more schools, saving educational costs and benefiting more teachers.

### 5.3. Limitations and Implications for Future Research

There were also some limitations in this study. First, our data consisted of primary school teachers, a female-dominated group; consequently, we cannot be sure that the same latent profiles of burnout and their transferring probabilities emerge in other groups of professionals. Therefore, the results of this study are applicable only to primary school teachers, and there is a need to replicate the profiles in the context of other professionals and to compare those groups with teachers in future studies. In addition, the study sample was from a district with strong educational level, whether the results of this study are applicable to other districts with relatively weak educational levels needs more comparative studies in the future. Second, the time-lags between measurements were not theoretically determined. Although a three-year time lag has been included in previous burnout studies [18], it is unknown whether this length of time best describes the burnout process among teachers. However, the temporal aspects of burnout are unclear, although some studies have suggested that burnout symptoms accumulate and develop over a long period [70]. In addition, there is some question as to whether the temporal aspects of burnout are related to occupation. This may be a question that needs to be answered once research on burnout development has been sufficiently enriched. Third, there are many factors affecting teacher burnout, including individual factors and situational factors (e.g., job-related factors, lifestyle and economic status, etc). The extension of the investigation of various individual or situational factors of teachers and the analysis of the main effect and coaction mechanism of these factors are necessary for future research. For example, this might include other risk factors or protective factors, and their possible interactions, that are in line with JD-R theory [31,32].

## 6. Conclusions

The present study used a person-centred approach in a longitudinal study on the development of teacher burnout and obtained certain valuable findings. There are six relatively stable latent profiles of teacher burnout and individual profile transfer to other profiles with a certain transition probability. Psychological capital and professional identity are important resource factors in reducing the occurrence of teacher burnout and increasing transition probability toward burnout symptom alleviation over time, while positive coping plays an important role in reducing the occurrence of teacher ineffectiveness. Our results emphasize the importance of understanding teacher burnout and its developmental process from the perspective of a combination of individual profile differences and overall profile consistency.

## Figures and Tables

**Table 1:** Fit statistics for latent profiles of teacher burnout at T1 and T2.

Wave	Number	AIC	aBIC	Entropy	*p*(LMR)	*p*(BLRT)	Probabilities of Each Profile
T1	1	24,810.80	24,828.25				1
	2	23,257.22	23,286.30	0.79	0.000	0.000	0.75;0.25
	3	22,522.68	22,563.40	0.81	0.000	0.000	0.58;0.14;0.28
	4	22,054.20	22,106.55	0.76	0.000	0.000	0.13;0.13;0.41;0.33
	5	21,666.62	21,730.60	0.78	0.000	0.000	0.41;0.06;0.20;0.18;0.15
	6	21,321.77	21,397.38	0.80	0.002	0.000	0.19;0.16;0.40;0.06;0.02;0.17
	7	21,148.52	21,235.76	0.81	0.042	0.000	0.36;0.08;0.06;0.10;0.17;0.19;0.03
	8	21,002.27	21,101.14	0.78	0.090	0.000	0.12;0.11;0.06;0.11;0.28;0.08;0.02;0.23
T2	1	23,888.97	23,906.42				1
	2	21,659.33	21,688.41	0.87	0.000	0.000	0.77;0.23
	3	21,016.51	21,057.22	0.87	0.000	0.000	0.25;0.71;0.04
	4	20,460.11	20,512.46	0.86	0.000	0.000	0.23;0.03;0.12;0.62
	5	20,120.98	20,184.95	0.83	0.006	0.000	0.57;0.08;0.17;0.03;0.14
	6	19,882.84	19,958.45	0.84	0.001	0.000	0.54;0.02;0.07;0.19;0.15;0.03
	7	19,670.32	19,757.57	0.78	0.045	0.000	0.26;0.08;0.02;0.07;0.38;0.13;0.06
	8	19,475.57	19,574.45	0.79	0.000	0.000	0.32;0.12;0.07;0.02;0.09;0.07;0.29;0.02

Note: aBIC = Bayesian information criterion calibrated by sample size; LMR = Lo-Mondell-Ruben’s calibrated likelihood ratio test; BLRT = Bootstrap likelihood ratio test.

**Table 2:** Mean of three dimensions in the six latent profiles.

Profiles	T1				T2			
	N	EE	DP	PA	N	EE	DP	PA
(1) Low/no burnout	1497(40%)	2.02	1.32	1.86	2021(54%)	1.92	1.32	1.79
(2) Highly ineffective	225(6%)	1.45	1.18	4.53	75(2%)	1.52	1.20	4.31
(3) Ineffective instigated	636(17%)	2.46	1.96	3.32	711(19%)	2.43	1.95	2.98
(4) Exhaustion instigated	711(19%)	3.50	1.78	2.15	262(7%)	3.73	1.79	1.88
(5) EE & DP dominated burnout	75(2%)	4.52	3.78	2.49	112(3%)	4.47	3.96	2.14
(6) Burnout	599(16%)	3.64	2.74	2.78	561(15%)	3.45	2.80	2.69
Mean (Total)		2.66	1.79	2.50		2.46	1.78	2.23
Median (Total)		2.56	1.67	2.25		2.22	1.67	2.00
*F* (5,3737)		1440.32	2967.14	1013.74		1341.77	3155.95	644.24
Effect size η^2^		0.690	0.821	0.610		0.674	0.830	0.498

**Table 3:** Latent transition probability of each profile from T1 to T2.

Latent Transition Probability		T2					
		(1)	(2)	(3)	(4)	(5)	(6)
T1	(1) Low/no burnout	62.0%	5.8%	22.5%	3.2%	0.8%	5.7%
	(2) Highly ineffective	60.1%	19.6%	6.8%	1.0%	0.5%	12.0%
	(3) Ineffective instigated	16.6%	21.3%	33.0%	1.9%	2.6%	24.6%
	(4) Exhaustion instigated	15.4%	1.9%	43.7%	27.0%	5.4%	6.5%
	(5) EE & DP dominated burnout	4.8%	7.0%	18.2%	16.5%	29.2%	24.3%
	(6) Burnout	14.2%	12.7%	23.9%	6.8%	11.5%	30.9%

**Table 4:** Means and standard deviations of resource factors in each profile at T1.

Profiles	Psychological Capital		Professional Identity		Positive Coping	
	M	SD	M	SD	M	SD
(1) Low/no burnout	3.31	0.36	4.20	0.76	3.31	0.47
(2) Highly ineffective	3.15	0.43	4.33	0.96	2.97	0.58
(3) Ineffective instigated	2.92	0.36	3.60	0.80	2.92	0.51
(4) Exhaustion instigated	3.07	0.37	3.60	0.82	3.22	0.46
(5) EE&DP dominated burnout	2.73	0.44	2.55	0.99	3.11	0.54
(6) Burnout	2.82	0.40	3.15	0.85	2.94	0.53
Total	3.13	0.39	3.84	0.85	3.16	0.50
F (5,3737)	267.92		268.22		91.67	
Effect size η^2^	0.292		0.293		0.124	

**Table 5:** The OR of the probability of each profile at T1 under the effects of resource factors.

Factors	Highly Ineffective	Ineffective Instigated	Exhaustion Instigated	EE & DP Dominated Burnout	Burnout	*p*
psychological capital	0.92	0.39 ***	0.45 ***	0.20 ***	0.26 ***	<0.001
professional identity	1.82 *	0.75 ***	0.68 ***	0.42 ***	0.55 ***	<0.001
positive coping	0.64 **	0.81	1.16	1.28	1.00	<0.01

Note: (1) * *p* < 0.05, ** *p* < 0.01, *** *p* < 0.001; (2) the *p*-value in the last column referred to the significance of the likelihood ratio test as a whole for each factor and *p* < 0.05 indicated that the factor had a significant influence on the outcome variables.

**Table 6:** The OR of transition from T1 to T2 with the effects of resilience and positive coping.

Latent Profile	Factors	(1)	(2)	(3)	(4)	(5)	(6)	*p*
(1) Low/no burnout	psychological capital	/	0.17 *	0.06 ***	0.18 ***	0.00 ***	0.01 ***	<0.001
	professional identity	/	12.26 ***	0.53 ***	0.38 ***	0.06 ***	0.19 ***	<0.001
	positive coping	/	0.70	0.52 ***	1.54	1.60	0.48 **	<0.001
(2) Highly ineffective	psychological capital	2.33	/	0.15 *	^a^	^a^	0.03 **	<0.001
	professional identity	0.77	/	0.66	^a^	^a^	0.25 **	<0.001
	positive coping	3.63 *	/	1.88	^a^	^a^	0.91	<0.05
(3) Ineffective instigated	psychological capital	21.58 ***	2.03	/	1.66	0.05 *	0.52 *	<0.001
	professional identity	2.47 ***	25.79 ***	/	1.05	0.22 ***	0.51 ***	<0.001
	positive coping	2.20 **	0.74	/	1.82	1.31	1.45	<0.01
(4) Exhaustion instigated	psychological capital	3.96 ***	1.65	0.17 **	/	0.00 ***	0.05 ***	<0.001
	professional identity	1.76 ***	1.97	1.06	/	0.14 ***	0.48 ***	<0.001
	positive coping	1.61	0.08 *	0.45 *	/	1.82	0.87	<0.001
(5) EE and DP dominated burnout	psychological capital	^a^	^a^	1.39	1.18	/	0.83	>0.05
	professional identity	^a^	^a^	3.42 *	5.71 **	/	1.37	<0.01
	positive coping	^a^	^a^	0.15 *	0.38	/	0.48	>0.05
(6) Burnout	psychological capital	24.84 ***	1.69	1.90	5.97 **	0.30 **	/	<0.001
	professional identity	4.99 ***	9.83 ***	2.40 ***	1.60 **	0.47 **	/	<0.001
	positive coping	2.37 **	0.47	0.54 **	2.36 **	2.36 **	/	<0.001

Note: (1) the rows were the latent profiles at T1, and the columns were the latent profiles at T2; (2) * *p* < 0.05, ** *p* < 0.01, *** *p* < 0.001; (3) ^a^ the number of individuals in this cell was less than five, the OR value was ignored; (4) the *p*-value in the last column referred to the significance of the likelihood ratio test as a whole for each factor and *p* < 0.05 indicated that the factor had a significant influence on the outcome variables.

## Data Availability

The data presented in this study are available on request from the corresponding author. The data are not publicly available due to privacy restrictions.
